# The Chemistry of Dental Caries

**Published:** 1890-10

**Authors:** R. C. Young

**Affiliations:** President of Alabama State Society.


ARTICLE IV.

### THE CHEMISTRY OF DENTAL CARIES.

BY R. C. YOUNG, D. D. S.

(President of Alabama State Society.)

Caries is a pathogenic condition of the teeth, in no way similar to caries of bone, except in name. Its pathology has for long ages been a subject of investigation. Up to the present time there has been no universally accepted theory. The ancients attributed it to some humor of the blood, and verily they were not far wrong. However, we will consider some of the more modern theories. He must indeed be fastidious who cannot be satisfied with such a *repetoire*. We have the chemical, chemico-vital, electro-chemical, infusorial or bacterial, and hereditary theories, each under the able pens of its advocates seeming as plausible and acceptable as the others.

Let us get at its etiology by elimination. "That the fathers have eaten sour grapes and the children's teeth are on edge" is, I think more metaphorical than practical. That one or both parents could transmit this disease of the teeth seems improbable. We can but admit that they do, beyond a doubt, transmit to the offspring a predisposition or a favoring condition, by not giving to the child a vitality sufficient to combat the cause of the effect. Should it be a transmitted heritage then caries must be a disease the germs of which are lurking in the blood, and our venerable father Æsculapius and his early deciples solved the problem.

Will you consider with me my favorite theory—chemico-vital? Did it ever occur to you, that the animal body is one vast chemical laboratory, and that through the mouth passes the material for the countless chemical actions and re-actions which are continually going on? What is the character of this material? Food must embody, two great principles, one to nourish, the other to supply heat to the body. In the process of digestion, these principles are separated into: 1st.—All that, whether animal or vegetable, which can be converted into albumen, and which is called albumenous, and constitutes nutritious matter: (we may add to these a few salts of the alkaline earths of which more anon). 2d.—All saccharine matters from vegetable or animal; these with the oleaginous principles are heat producers and are known as calorifacients.

Chemical analysis will demonstrate that every article from which these principles can be derived is convertible into acids which act directly upon the teeth. All soft or plastic matter taking on formation must have some skeleton or frame. The bones give this support. It is otherwise with the teeth. They have their origin as a gelatinous mass whose form and contour are hardened and kept in shape by an infiltration of salts. I do not believe that here the animal and mineral are in what we understand to be a chemical union or solution, but rather a mechanical mixture. Let me illustrate my meaning by the simplest demonstration possible. Take water. Here we have a chemical union between oxygen and hydrogen, which can only be separated into its elements by a force equal to the force which was developed in their union. On the other hand the air which we breathe is simply a mixture, the oxygen and nitrogen being in no way chemically united. Thus they are separated with but little force.

We have said that all food passing through the mouth can be converted into acids. The inquiring mind will naturally ask how? Chiefly through fermentation. This name has been given to an interesting class of chemical changes which occur in all organic matter under favoring conditions, which conditions the mouth present *non cum exceptionibus*, and which are the primary steps of decomposition: different matter giving rise to different products, but all acid. The following are some of the principle forms. Alcohol fermentation producing $CO_2$, carbonic acid, and as regards the teeth probably the most harmless; the actous fermentation producing chiefly $C_2H_4O_2$, acetic acid. The lactic yielding lactic acid, $C_3H_6O_3$. Through its affinity for calcium this acid is present in all animal flesh. It is also a product of saccharine fermentation. We find it present in the repair of tissue and it may from its numerous sources be looked upon as the many-headed dragon. Remember the large amount of sugar entering into the *regime* of our daily diet, not only as sugar proper, but by the action of ptyline upon starch or farinaceous matter.

Other acids, we have, which are not of this fold, HCL, hydro-chloric, $H_2SO_4$, sulphuric, $HNO_3$, nitric, which last in my opinion is next to lactic in its deleterious effects upon the teeth. In fact which of these two acids is the prime factor in that most deplorable of all dental lesions, white decay, is an unsolved question to me. This class of decay must be associated either with patients suffering from chronic gingivitis, or with mouth breathers. In the former, pathology teaches that lactic acid is always present where granulation is going on. Take a patient with chronically inflamed gum margins and I guarantee you will find cavities of white decay, with hyperæsthesia, upon or about the cervix of the teeth. In those who breathe through the mouth almost the same condition will be found not however so strictly confined to the same territory. May not this be the effect of nitric acid, from the free supply of air oxidizing the nitrogenous matter present in the mouth? But you say you find this condition in no mouths which are kept scrupulously clean. So you may. $HNO_3$, nitric acid, is formed by oxidation of nitrogenous matter. In the presence of potassa we have a free supply of oxygen from the air. The saliva furnishes both the nitrogenous matter and potassa, the former the epithelium and soluble organic matter which by evaporation of the water of saliva are condensed. Admitting these acids to be present and knowing the teeth to be largely composed of lime salts the presence of caries seems readily accounted for.

All hydrogen salts when brought in contact with a suitable base form some soluble salt. Here we have them searching out the lime from the teeth, exposing the now unprotected animal matter which breaks down by first undergoing fermentation, forming more acid *per se*, then decomposition ; in come the bacteria. Here is nutriment for them. Here they are in countless numbers feeding on this decomposed animal matter—removing each layer as it is formed by this affinity for acid for base. Step by step, atom by atom, continues the silent destruction of these faithful sentinals which guard the portal of life, wrecking health, happiness and beauty.

What is more indicative of health than a perfect set of teeth? Where is happiness without health? Do they both dwell in the same temple? Need I ask what is more beautiful, what more expresive than those pearly gems peeping from rosy lips which part in words of love and sympathy, or wreath themselves into smiles sweeter and more welcome than the chaplet to the conquering hero?

But let us return. We are confronted by a problem of apparent difficulty. We have found the product of fermentation to be acid. Not so with that of putrefaction. Here we are met by alkaloids which are not acid. Why then should they not neutralize the acid of fermentation and check the chemical action, *per se*, thus giving nature a chance to come to the rescue and repair the lesion as under favorable conditions she does in soft tissues? Will you agree with me when I say this does occur? Do we not see teeth which have decayed when with no interference of art the disease is arrested? May not this be brought about by re-action, the products of decomposition neutralizing those of fermentation giving nature an opportunity to exercise her functions? The vital forces needed in such cases is great. We can readily picture to ourselves patients in whose mouths this condition will be found. Are they not always disciples of Hygeia? Take a case in which this retrogressive metamorphosis has been checked. We find the bottom of the cavity to be vitrified dentine, corresponding to cicatricial tissue. Is this not a clear indication of what the great healer nature would have done? Let us accept from her our rule, and with the helping of science the day will not be far off when we can proudly unfurl our banner, on which suffering humanity may read "VICTRIX."
—*Dental Mirror.*

